# Non‐physician delivered intravitreal injection service: A systematic review of safety, implementation, training and patient experience

**DOI:** 10.1111/aos.70104

**Published:** 2026-02-11

**Authors:** Trang Truong Laursen, Haris Bralic, Jakob Grauslund, Yousif Subhi, Lonny Stokholm

**Affiliations:** ^1^ Department of Ophthalmology Odense University Hospital Odense Denmark; ^2^ Department of Clinical Research University of Southern Denmark Odense Denmark; ^3^ Department of Regional Health Research University of Southern Denmark Odense Denmark; ^4^ Department of Ophthalmology Vestfold Hospital Trust Tønsberg Norway; ^5^ Department of Ophthalmology University Hospital of Southern Denmark Sønderborg Denmark; ^6^ Department of Clinical Medicine University of Copenhagen Copenhagen Denmark

**Keywords:** anti‐VEGF, intravitreal injection, patient experience, safety, systematic review, task shifting

## Abstract

The global demand for intravitreal anti‐vascular endothelial growth factor (anti‐VEGF) therapy continues to rise, straining ophthalmic capacity worldwide. Task shifting from physicians to trained non‐physician healthcare professionals has emerged as a potential strategy to expand service delivery. This systematic review summarizes current evidence on the safety, implementation, training and patient experiences associated with non‐physician‐delivered intravitreal injection services. We systematically searched 13 scientific literature databases for studies of anti‐VEGF injections performed by non‐physicians. Data were extracted on safety outcomes, implementation, training, supervision and patient‐reported experience. Results from individual studies were qualitatively reviewed according to these four organizational themes. Sixteen studies comprising 100 150 injections were included, primarily from the United Kingdom and Scandinavia. All injectors completed structured training under an ophthalmologist's supervision. Complication rates were very low, including 0.015%–0.07% for endophthalmitis which were comparable to physician‐led benchmarks. Implementation of non‐physician‐delivered intravitreal injection services increased clinical capacity by 25%–85%, reduced waiting times and costs, and maintained safety and efficiency. Patients reported high satisfaction, confidence and acceptance of non‐physician‐delivered injections. Non‐physician‐delivered intravitreal injection services are safe, feasible and well accepted when supported by structured training and robust governance frameworks. This task‐shifting model offers a scalable solution to meet growing global demand for anti‐VEGF therapy. Standardized international training and certification frameworks may further enhance safety and sustainability.

## 1 INTRODUCTION

Intravitreal therapy with vascular endothelial growth factor (anti‐VEGF) agents has revolutionized the treatment of retinal diseases such as neovascular age‐related macular degeneration (nAMD), diabetic macular oedema (DME) and retinal vein occlusion (RVO) (Pham et al., 2019; Schmidt‐Erfurth et al., 2014; Wong et al., 2014). Due to the chronic and recurrent nature of these conditions, anti‐VEGF treatment is often required over many years, placing a considerable burden on healthcare systems (Lin et al., 2021). The global prevalence of age‐related macular degeneration is projected to increase from 196 million in 2020 to 288 million by 2040, driven by population ageing and expanded treatment eligibility (Wong et al., 2014). In Europe, the number of individuals affected by nAMD is projected to rise substantially from approximately 67 million in 2020 to nearly 109 million by 2040. In addition to nAMD, both DME and RVO contribute substantially to the global burden of retinal disease (Teo et al., 2020). DME is estimated to affect approximately 4%–7% of individuals with diabetes worldwide, while RVO has a global prevalence of around 0.77% among adults aged 30–89 years, corresponding to nearly 28 million people (Heloterä et al., 2024; Song et al., 2019).

Because these conditions require long‐term, repeated intravitreal anti‐VEGF treatment, the rising epidemiological burden translates directly into increasing demand for treatment capacity (Michelotti et al., 2014). Consequently, ophthalmology services—particularly in publicly funded health systems with limited specialist availability—are facing increasing constraints on clinic capacity and timely access to care (Teo et al., 2020).

The World Health Organization (WHO) has identified task shifting as the delegation of specific clinical responsibilities from physicians to trained non‐physician health professionals as a strategy to expand healthcare capacity safely and sustainably (World Health Organization et al., 2008).

In line with these global principles, several countries have adopted task‐shifting models that allow non‐physician healthcare professionals, primarily nurses, to perform anti‐VEGF injections under structured training and supervision (Bolme et al., 2020; Rasul et al., 2016; Samalia et al., 2016; Simcock et al., 2014; Teo et al., 2020). Although the global prevalence of nurse‐administered intravitreal injections has not been systematically quantified, reports from Europe, New Zealand, and Asia indicate a growing adoption of nurse‐led injection services in response to increasing treatment demand (Bolme et al., 2020; Hasler et al., 2015; Michelotti et al., 2014; Teo et al., 2020). Initial evaluations suggest that anti‐VEGF treatment delivered by trained non‐physicians is feasible, safe and well accepted by patients, while contributing to improved service efficiency and reduced waiting times (Teo et al., 2020; Varma et al., 2013). However, variation exists in how these models are implemented across regions, particularly with respect to training, clinical governance and patient communication (Rasul et al., 2016).

The first systematic review on non‐physician‐administered anti‐VEGF treatment identified five studies involving more than 31 000 injections and concluded that this model was safe and feasible (Rasul et al., 2016). However, the evidence base was limited at the time, and the authors recommended further research to assess broader outcomes, including patient experience, clinical effectiveness and implementation challenges (Rasul et al., 2016). Since then, the number and scope of such programmes have expanded considerably. As ophthalmic services worldwide face growing capacity constraints, understanding the evidence surrounding non‐physician‐administered intravitreal therapy has become increasingly important. This systematic review aims to summarize current evidence on the safety, implementation, training, supervision and patient experiences associated with non‐physician‐delivered anti‐VEGF treatment.

## 2 MATERIALS AND METHODS

### 2.1 Study design

This study is a systematic review designed in accordance with the Cochrane Handbook for Systematic Reviews of Interventions (Higgins et al., 2024). The protocol was prospectively registered in the PROSPERO database (registration number; CRD420251148449). The dissemination of the systematic review was made in accordance with the PRISMA 2020 (Preferred Reporting Items for Systematic Reviews and Meta‐Analyses) guidelines (Higgins et al., 2024; Moher et al., 2009). This systematic review required no ethical approval under Danish legislation. Ethical compliance in the included studies was assessed based on their reporting; where stated, studies indicated adherence to the Declaration of Helsinki, although such reporting was inconsistent.

### 2.2 Study eligibility

We included studies in which intravitreal anti‐VEGF injections were administered by non‐physician healthcare professionals, including but not limited to nurses, optometrists and orthoptists. The study populations comprised adults receiving anti‐VEGF treatment for any retinal disease, including nAMD, DME, and RVO. Outcomes of interest were safety, implementation strategies, training models and patient experiences. Both quantitative and qualitative study designs were considered eligible, including observational, interventional and interview‐based studies. We did not consider studies without original data, conference abstracts, non–peer‐reviewed material or case reports to be eligible. For practical purposes, we only included studies published in English.

### 2.3 Information sources, search strategy and study selection

A systematic literature search was conducted in *PubMed, Embase, Cochrane Central, BIOSIS Previews, Current Contents Connect, Data Citation Index, Derwent Innovations Index, KCI‐Korean Journal Database, Preprint Citation Index, ProQuest™ Dissertations & Theses Citation Index, SciELO Citation Index, Web of Science Core Collection and CINAHL*. The systematic literature search was performed by one trained author (Y.S.) on 14 September 2025. Details of the search strategy is available as File S1.

All extracted references were imported into EndNote 21.4 (Bld 18 113) for reference management. One author (T.T.L.) examined the titles and abstracts of all identified records, removed duplicates and excluded records that were obviously irrelevant based on title and abstract. Remaining references were obtained in full‐text for eligibility evaluation. Two authors (T.T.L. and H.B.) independently evaluated full‐text studies for potential eligibility. Any disagreements were resolved through discussion, and if consensus was not reached, a third author (L.S.) was consulted for final decision‐making.

### 2.4 Data collection

Data were extracted using a standardized form developed a priori. For each study, information was extracted on study design and context (year of publication, country/site, design, setting), population (number of patients, diagnosis, indication), intervention (type of provider, training and supervision, number of injections), safety outcomes (frequency and type of complications), implementation aspects (organization, governance and guidelines) and patient‐related outcomes (experiences, satisfaction, acceptance and waiting time). Two authors (T.T.L. and H.B.) independently extracted data from studies. Any disagreements were resolved through discussion, and if consensus was not reached, a third author (L.S.) was consulted for final decision‐making.

### 2.5 Risk of bias within study assessment

Risk of bias within individual studies was assessed using the appropriate Critical Appraisal Skills Programme (CASP) checklist for each study design (e.g. CASP Cohort, CASP Cross‐Sectional, CASP Randomized Controlled Trial (RCT), CASP Qualitative, CASP Economic Evaluation). Relevant items from the Agency for Healthcare Research and Quality (AHRQ) checklist were additionally consulted for descriptive cross‐sectional studies. Two reviewers (T.T.L. and H.B.) independently conducted the appraisal, with disagreements resolved through discussion or, if necessary, by a third reviewer (L.S.).

### 2.6 Synthesis of results

All studies were qualitatively reviewed and described in text and in tables. The qualitative review was organized into four predefined thematic domains: safety, implementation, training and supervision, and patient‐reported experiences.

## 3 RESULTS

**FIGURE 1:**
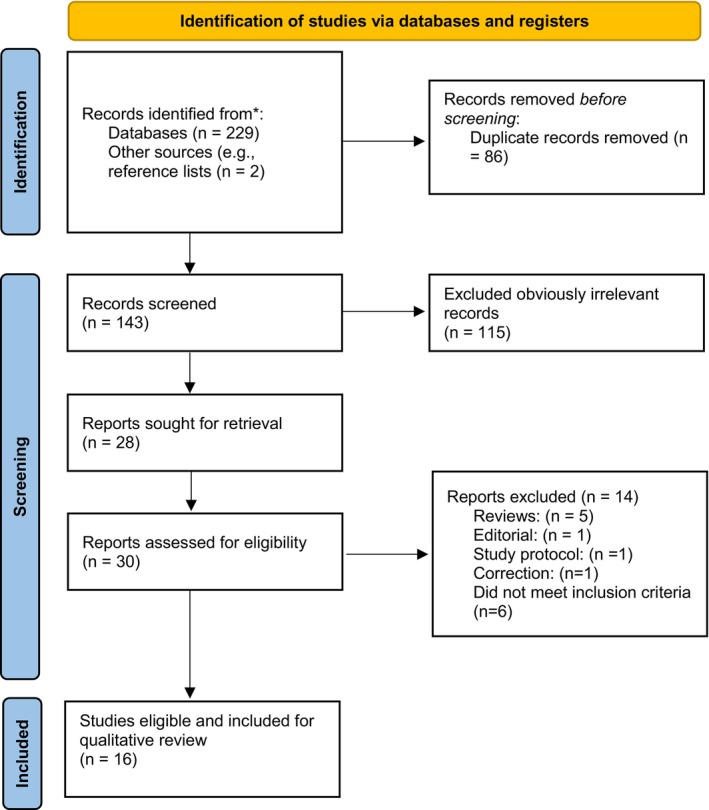
PRISMA 2020 flow diagram for new systematic reviews which included searches of databases and registers only.

The literature search identified 229 records. After removal of duplicates (*n* = 86), 143 articles remained for screening. Of these, 115 were excluded based on title and abstract, leaving 28 articles for full‐text assessment. Two additional studies were identified through reference searching, resulting in 30 full‐text articles assessed for eligibility. In total, 16 studies were included in the final synthesis, as illustrated in Figure 1.

The 16 included studies comprised 100 150 intravitreal injections performed by trained non‐physician injectors, all of whom were ophthalmic nurses. Most studies originated from the United Kingdom.

(*n* = 9), followed by Norway (*n* = 3), Denmark (*n* = 2), Singapore (*n* = 1) and New Zealand (*n* = 1) (Ahmed et al., 2022; Bolme et al., 2020, 2021, 2023; Chakouri et al., 2017; DaCosta et al., 2014; Gallagher, 2017; Hasan et al., 2017, 2020; Hasler et al., 2015; Michelotti et al., 2014; Mohamed et al., 2018; Samalia et al., 2016; Simcock et al., 2014; Teo et al., 2020; Varma et al., 2013).

The evidence base consisted of 16 studies: two randomized controlled trials (Bolme et al., 2020; Mohamed et al., 2018), three prospective studies (Bolme et al., 2021, 2023; Gallagher, 2017), eight prospective cohort studies (Ahmed et al., 2022; DaCosta et al., 2014; Hasan et al., 2017; Michelotti et al., 2014; Samalia et al., 2016; Simcock et al., 2014; Teo et al., 2020; Varma et al., 2013), one retrospective audit (Hasan et al., 2020), and one retrospective cohort study (Hasler et al., 2015), and one correspondence article containing original data (Chakouri et al., 2017).

Across all studies, nurses underwent structured training and supervision, performing between 20 and 200 supervised injections before certification. The majority of programmes were implemented in tertiary ophthalmology units under ophthalmologist governance. A summary of study characteristics is available in Table 1.

**TABLE 1:** Characteristics of included studies on non‐physician‐administered intravitreal anti‐VEGF injections.

References	Country	Design	Setting	Non‐physician role	Supervised injections, (*n*)	Reported domains	No. of injections (*n*)
Ahmed et al. (2022)	United Kingdom	Respective Cohort study	Hospital eye Department	Ophthalmic nurses, 3 → 7	50 supervised injections	Safety, device effect, cost	20 421
Bolme et al. (2020)	Norway	Prospective RCT study	Hospital eye Department	Ophthalmic nurses (6 trained)	100 supervised	Safety, visual outcomes, satisfaction	1076
Bolme et al. (2021)	Norway	Prospective Qualitative Study	Hospital eye Department	12 nurses interviewed	Supervised during training	Nurse perspectives, confidence, workflow	NR
Bolme et al. (2023)	Norway	Prospective study	Hospital eye Department	Nurses per trial protocol	Supervised during training	Cost, efficiency, resource use	NR
Chakouri et al. (2017)	Denmark	Respective Correspondances	Hospital eye Department	Ophthalmic nurses with operating backgrounds	4 weeks of supervision	Safety, training, implementation, patient acceptance	5817
DaCosta et al. (2014)	United Kingdom	Cohort study	Hospital eye Department	Band 7 nurse practitioners	~100 supervised	Safety, satisfaction, capacity, governance	4000
Gallagher (2017)	United Kingdom	Prospective Cross‐sectional studies	Hospital eye Department	1 advanced ophthalmic nurse practitioner, MSc level	50 observed + 50 supervised	Patient survey, governance, training	NR
Hasan et al. (2017)	United Kingdom	Prospective Cross‐sectional studies	Hospital eye Department	5 nurses	≥50 supervised	Patient experience, anxiety, satisfaction	NR
Hasan et al. (2020)	United Kingdom	Cohort study	Hospital eye Department	8 independent nurse injectors	≥50 supervised (50 observed + 50 supervised)	Safety, training, efficiency, cost	>30 000 (2014–2019)
Hasler et al. (2015)	Denmark	Retrospective Cohort study	Hospital eye Department	4 ophthalmic nurses, stepwise training	8–10 supervised model practice	Injector mix, complications	12 542
Michelotti et al. (2014)	United Kingdom	Prospective Cohort study	Hospital eye Department	Experienced ophthalmic nurses. Hospital A trained 3 nurses sequentially	First 200 supervised	Safety, patient preference, efficiency, cost	3355
Mohamed et al. (2018)	United Kingdom	Prospective RCT study	Central Middlesex Hospital	Trained clinical nurse	NR	Patient satisfaction, pain score, complications	34
Samalia et al. (2016)	New Zealand	Prospective cohort study	Hospital eye Department	3 nurse specialists with operating backgrounds	~50 supervised	Safety, training, governance	2900
Simcock et al. (2014)	United Kingdom	Prospective Cohort study	Hospital eye Department	Nurse practitioners with surgical ophthalmic experience	First 20 supervised	Complications, governance, consent	10 006
Teo et al. (2020)	Singapore	Retrospective cross‐sectional study	Hospital eye Department	Registered nurses, credentialed, supervised training	First 100 supervised	Safety, satisfaction, waiting time, cost	8599
Varma et al. (2013)	United Kingdom	Prospective and retrospective Cohort study	Hospital eye Department	4 Specialist nurses, Band 7, surgical background	25 intravitreal injections	Safety, patient pain, satisfaction, governance	1400

Abbreviations: *n*, number; NR, not reported; RCT, randomized controlled trial.

In our risk of bias assessment, most studies clearly reported study context, eligibility criteria and training procedures, ensuring acceptable transparency. Prospective and implementation studies provided detailed descriptions of nurse training and supervision, whereas some retrospective audits reported limited information on data completeness. Blinding was largely not applicable due to the service delivery design of most studies; in the single RCT, patient masking was only partially successful. Potential selection bias was noted where nurses treated predominantly low‐complexity patients. Across the included studies, risk of bias was generally rated as low to moderate and acceptable for this review, as presented in Tables 2, 3, 4, 5, 6. A summary of safety, implementation, training and patient‐reported experience for each study is presented in Table 7.

**TABLE 2:** Risk of bias assessment – cohort studies.

Cohort study checklist	Ahmed et al. (2022)	DaCosta et al. (2014)	Hasan et al. (2020)	Hasler et al. (2015)	Michelotti et al. (2014)	Samalia et al. (2016)	Simcock et al. (2014)	Varma et al. (2013)
1. Did the study address a clearly focused issue?	Yes	Yes	Yes	Yes	Yes	Yes	Yes	Yes
2. Was the cohort recruited in an acceptable way?	Yes	Yes	Yes	Yes	Yes	Yes	Yes	Yes
3. Was the exposure accurately measured to minimize bias?	Yes	Yes	Yes	Yes	Yes	Yes	Yes	Yes
4. Was the outcome accurately measured to minimize bias?	Yes	Yes	Yes	Yes	Yes	Yes	Yes	Yes
5. (a) Have the authors identified all important confounding factors	Can't tell	Can't tell	No	No	No	Can't tell	Can't tell	No
(b) Have they taken account of the confounding factors in the design and/or analysis?	Can't tell	No	No	No	No	No	No	Yes
6. (a) Was the follow up of subjects complete enough?	Can't tell	Yes	Yes	Yes	Yes	Yes	Can't tell	Can't tell
(b) Was the follow up of subjects long enough?	Yes	Yes	Yes	Yes	Yes	Yes	Yes	Can't tell
7. What are the results of this study?	Large sample size (26 923 injections) with robust complication tracking	Rigorous training and governance; >4000 injections with no serious events. Continuous audit and supervision	Nurses delivered over 85% of >30 000 injections with complication rates matching national data.	38 503 injections were delivered with a 0.44‰ complication rate and no difference in endophthalmitis rates between nurses and trainee ophthalmologists	The nurse‐led model increased capacity, enabled one‐stop care, maintained high patient satisfaction, reduced reliance on ophthalmologists, and lowered costs	Nurse specialists safely administered intravitreal anti‐VEGF injections, increasing service capacity, improving access, freeing ophthalmologist time, and reducing costs	Nurses delivered 10 006 ranibizumab injections over 5.5 years with a very low endophthalmitis rate (0.04%) and no lens injuries or retinal detachments, confirming a strong safety profile	In 1400 nurse‐delivered intravitreal injections, minor complications (1.4%) occurred, no serious adverse events were reported, pain scores were minimal, and outcomes matched ophthalmologist‐led care
8. How precise are the results?	Can't tell	Can't tell	Can't tell	Yes	Yes	Can't tell	Can't tell	Yes
9. Do you believe the results?	Yes	Yes	Yes	Yes	Yes	Yes	Yes	Yes
10. Can the results be applied to the local population?	Yes	Yes	Yes	Yes	Yes	Yes	Yes	Yes
11. Do the results of this study fit with other available evidence?	Yes	Yes	Yes	Yes	Yes	Yes	Yes	Yes
12. What are the implications of this study for practice?	The nurse‐led Precivia®‐assisted IVT service was safe, cost‐effective, increased capacity, and maintained a very low endophthalmitis rate (0.015%) with high patient satisfaction	Nurse‐led intravitreal injection services are documented to be safe, feasible, and a sustainable strategy for increasing capacity in medical retina clinics	The Swindon model demonstrated that nurse‐led IVT services with structured training and Precivia® support were safe, efficient, and cost‐effective in meeting rising demand	Nurse‐led intravitreal injections were safe when delivered under structured training, standardized protocols, and theatre conditions, with extremely low complication rates	Trained non‐physician staff safely delivered IVT injections, improving efficiency, reducing wait times, and freeing ophthalmologists to enhance retinal care delivery	Trained nurse specialists safely administered anti‐VEGF injections, increased service capacity, freed ophthalmologist time, improved access, and reduced per‐treatment costs	The study shows that carefully selected and well‐trained nurses can safely and effectively deliver wAMD intravitreal injections, offering a viable service model to relieve growing National Health Service demand for anti‐VEGF treatment	Nurse‐delivered intravitreal injections offer a clinically and cost‐efficient way to meet rising demand, improving productivity while maintaining quality, given robust training and clinical governance

**TABLE 3:** Risk of bias assessment – cross‐sectional studies.

Cross‐sectional studies checklist	Gallagher (2017)	Hasan et al. (2017)	Teo et al. (2020)
1. Did the study address a clearly focused issue?	Yes	Yes	Yes
2. Did the authors use an appropriate method to answer their question?	Yes	Yes	Yes
3. Were the subjects recruited in an acceptable way?	No	Yes	Yes
4. Were the measures accurately measured to reduce bias?	Yes	Yes	Yes
5. Were the data collected in a way that addressed the research issue?	Yes	Yes	Yes
6. Did the study have enough participants to minimize the play of chance?	Can't tell	Can't tell	Yes
7. How are the results presented and what is the main result?	Yes	Yes	Yes
8. Was the data analysis sufficiently rigorous?	Yes	Yes	Yes
9. Is there a clear statement of findings?	Yes	Yes	Yes
10. Can the results be applied to the local population?	Yes	Yes	Yes
11. How valuable is the research?	Supports safe, high‐satisfaction nurse‐delivered care as a capacity‐enhancing solution, consistent with previous evidence	Robust description of training and implementation, strong patient acceptability	Strong evidence that nurse‐led intravitreal injections are safe, efficient, and acceptable in a large tertiary centre in Singapore.

**TABLE 4:** Risk of bias assessment – randomised controlled trials.

Randomized controlled trial checklist	Bolme et al. (2020)	Mohamed et al. (2018)
1. Did the study address a clearly formulated research question?	Yes	Yes
2. Was the assignment of participants to interventions randomized?	Yes	Yes
3. Were all participants who entered the study accounted for at its conclusion?	Yes	Yes
4. (a) Were the participants ‘blind’ to intervention they were given?	Yes	No
(b) Were the investigators ‘blind’ to the intervention they were giving to participants?	No	No
(c) Were the people assessing/analysing outcome/s ‘blinded’?	Can't tell	Can't tell
5. Were the study groups similar at the start of the randomized controlled trial?	Yes	Can't tell
6. Apart from the experimental intervention, did each study group receive the same level of care (that is, were they treated equally)?	Yes	Yes
7. Were the effects of intervention reported comprehensively?	Yes	Yes
8. Was the precision of the estimate of the intervention or treatment effect reported?	Yes	No
9. Do the benefits of the experimental intervention outweigh the harms and costs?	Yes	Yes
10. Can the results be applied to your local population/in your context?	Yes	Yes
11. Would the experimental intervention provide greater value to the people in your care than any of the existing interventions?	Yes	Yes

**TABLE 5:** Risk of bias assessment – qualitative studies.

Qualitative studies checklist	Bolme et al. (2021)
1. Was there a clear statement of the aims of the research?	Yes
2. Is a qualitative methodology appropriate?	Yes
3. Was the research design appropriate to address the aims of the research?	Yes
4. Was the recruitment strategy appropriate to the aims of the research?	Yes
5. Was the data collected in a way that addressed the research issue?	Yes
6. Has the relationship between researcher and participants been adequately considered?	Yes
7. Have ethical issues been taken into consideration?	Yes
8. Was the data analysis sufficiently rigorous?	Yes
9. Is there a clear statement of findings?	Yes
10. How valuable is the research?	Yes

**TABLE 6:** Risk of bias assessment – economic evaluation.

Economic evaluation checklist	Bolme et al. (2023)
1. Was a well‐defined question posed?	Yes
2. Was a comprehensive description of the competing alternatives given?	Yes
3. Does the paper provide evidence that the programme would be effective?	Yes
4. Were the effects of the intervention identified, measured and valued appropriately?	Yes
5. Were all important and relevant resources required, and health outcome costs for each alternative identified, measured in appropriate units and valued credibly?	Yes
6. Were costs and consequences adjusted for different times at which they occurred (discounting)?	Can't tell
7. What were the results of the evaluation?	Yes
8. Was an incremental analysis of the consequences and cost of alternatives performed?	Yes
9. Was an adequate sensitivity analysis performed?	Yes
10. Is the programme likely to be equally effective in your context or setting?	Yes
11. Are the costs translatable to your setting?	Can't tell
12. Is it worth doing in your setting?	Yes

**TABLE 7:** Summary of safety, implementation, training and patient‐reported outcomes across nurse‐led intravitreal injection studies.

References	Safety	Implementation	Training and supervision	Patient‐reported experience
Ahmed et al. (2022)	Endophthalmitis 0.15 per 1000 injections	The task shifting involved nurses replacing physicians (an increase from 49.9% to 87.7% of injections performed by nurses) using the Precivia assist device. The device is cost‐effective and improves patient positioning	Nurses underwent a 12‐week training programme from induction to independent injection. The number of injectors increased from 3 to 7 over a 5‐year period	A high standard of patient satisfaction was maintained
Bolme et al. (2020)	Ocular adverse events occurred in 3 of the 1076 nurse‐administered injections, corresponding to an incidence of 2.8 per 1000 injections (0.28%)	Task shifting was implemented in routine practice with patients randomized to nurse or physician groups	Training followed a structured program with at least 20 supervised procedures and ophthalmologist evaluation before independence. Nurses were trained by a vitreoretinal surgeon, with annual refresher courses and re‐certification. A physician was always available during procedures	RCT: patient satisfaction equivalent between groups
Bolme et al. (2021)	NR	Implementation involved focus on collaboration, task distribution, and adaptation of workflows. Barriers included the need for clearly defined responsibilities and continuous adjustment of training and clinical routines as experience accumulated	Training programme: 10 days including workshops, wet lab sessions, observation, and supervision. Nurses are trained, certified, and undergo annual re‐certification. A designated physician is appointed to provide clinical guidance and ensure ongoing quality assurance. Nurses preferred supervision by experienced peers rather than by physicians	Qualitative nurse perspectives; no patient survey
Bolme et al. (2023)	NR	Patients (*n* = 318) were randomized to either physician‐ or nurse‐administered injections, and data were prospectively collected	The final version of the training programme lasts 10 days and comprises workshops, wet lab, and observation before nurses perform injections	NR
Chakouri et al. (2017)	No serious adverse events (no endophthalmitis or retinal detachment)	Implemented through literature review, thorough preparation, and organizational collaboration; nurses were trained and worked independently with physicians available for complex cases	Systematic training programme: 6‐h theoretical course, practical training on model eyes, study visit to Moorfields, 4 weeks of supervised injections, and certification before independent practice	Patients were generally satisfied; none refused nurse‐administered injections, and no patient concerns were reported.
DaCosta et al. (2014)	Subconjunctival haemorrhage occurred in 228 of 4000 injections (57.0‰), while no sight‐threatening adverse events such as endophthalmitis, retinal detachment, cataract, loss of central artery perfusion, vitreous haemorrhage or uveitis	Implementation included legal clarification, dedicated resources, a pilot project, and continuous audit under a responsible consultant. The main barrier was nurse licensing legislation, but the initiative was approved and scaled across sites	Rigorous training: One‐day course (theory + wet lab), ≥20 observed injections, pig‐eye practice and ≥100 supervised procedures before certification. Continuous supervision, specialist access and ongoing professional development ensured	Survey; patients satisfied with nurse injections
Gallagher (2017)	NR	The task shifting was implemented through an expansion of nurses' roles to include IVT, supported by local and national guidelines, master's‐level education, and quality management. Ensuring sufficient logistical and clinical capacity was prioritized	Nurses were required to complete theoretical training, observe 50 procedures, participate in practical wet lab sessions, undergo competency assessment, and perform 50 IVTs under supervision before certification. Continuous supervision and annual audits were implemented, with specialist support available at all times	100% satisfied; 81% not concerned about nurse versus doctor; 15% preferred nurse, 4% preferred doctor
Hasan et al. (2017)	NR	A dedicated treatment room was established with standardized procedures using the InVitria device and a team structure (1 injector +2 assistants). Patient flow followed local and national guidelines, with quality assurance through continuous patient feedback and surveys	Senior nurses, specially trained in the procedure and device use, worked under local protocols with access to medical supervision. Training included hands‐on practice, assistive equipment use and physician guidance	Survey; reduced anxiety; majority satisfied
Hasan et al. (2020)	Complication rates among >30 000 nurse‐administered injections were comparable to national data and physician‐delivered treatment	The implementation of nurse‐led intravitreal injection clinics began at Great Western Hospital in June 2015, after insurance and reimbursement conditions were met	Implemented via a 12‐week training programme using the Precivia device, reducing costs by 50% and increasing capacity from 13 to 17 patients per session, supported by clear roles and organization	Survey; high satisfaction, preference for nurse service
Hasler et al. (2015)	Endophthalmitis occurred in 14 of 38 503 injections, corresponding to an incidence of 0.36‰	The injection procedure and perioperative procedures were standardized and protocolized. All intravitreal injections were made in operating rooms	Trained for the purpose by vitreoretinal surgeons. Training included injections on an eye model and then 8–10 injections performed under direct supervision	NR
Michelotti et al. (2014)	Minor adverse events occurred in 12 of 3355 nurse‐administered injections, corresponding to an incidence of 3.6‰	Dedicated rooms were established or refurbished. Nurse‐led injections rapidly became the dominant model, enabling “one‐stop” clinics, reduced waiting times, and freed specialist capacity	Intensive training programme: nurses observed over 1000 procedures, performed the first 200 under direct specialist supervision, and underwent final competency assessment by a retinal consultant. After certification, nurses operated independently with a supervising ophthalmologist available on‐site	Improved patient satisfaction due to better service access, evidenced by formal and informal feedback and fewer complaints
Mohamed et al. (2018)	Nurse‐led intravitreal injection delivery achieved comparable safety, visual outcomes, and complication rates to consultant‐led care. No complications or patient complaints were recorded during the study period	Intravitreal injection is the most frequently performed ophthalmic procedure. Nurse‐led delivery was implemented to increase capacity and prevent vision loss	The study involved a trained clinical nurse. The authors recommend updating national guidelines to formally recognize the role of specialized and advanced practice nurses in delivering high‐quality care	Validated patient questionnaire and pain scale data showed no significant difference in patient satisfaction or pain between injections administered by a trained nurse and a senior ophthalmologist
Samalia et al. (2016)	A total of 9 adverse events were reported among 2900 injections (3.1‰), including an endophthalmitis incidence of 0.7‰	Nurse injector scheme launched 2013 in a tertiary ophthalmology clinic, with clinics running parallel to medical clinics for safety. Nurses handled 84% of injections by year two. Senior consultant maintained clinical oversight; workflow optimized capacity and cost‐effectiveness	Nurses underwent supervised training (minimum 50 observed/proctored injections), standard training package and hands‐on evaluation, followed by independent work after demonstrated competency. Continuous audit and required departmental and regulatory approvals	High patient acceptance and feeling safe
Simcock et al. (2014)	Endophthalmitis occurred in 4 of 10 006 injections, corresponding to an incidence of 0.40‰, with no cases of lens touch, retinal detachment, or systemic thromboembolic events reported	The task‐shift was introduced due to shortages of physicians and space. Designated nurse practitioners (NP) with surgical experience were trained and worked under supervision. A dedicated injection room was established. Continuous audit and governance procedures were implemented. The Clinical Governance Committee (CGC) approved the task‐shift and assumed responsibility	Selected NPs had prior experience in minor surgery or anaesthesia and were trained one‐to‐one by a vitreoretinal surgeon. A minimum of 20 supervised injections was required before independent practice. A supervising consultant was present during all NP‐administered injections, with ongoing audit and evaluation	NR
Teo et al. (2020)	Subconjunctival haemorrhage occurred in 4% of injections (40‰), intravitreal air in 0.55%, and one case of corneal abrasion was reported; no endophthalmitis occurred	Implemented at a national centre with university‐educated nurses only. Close collaboration between management and senior physicians. Parallel clinic operation ensured backup for adverse events. Patients received information via video and leaflet prior to the procedure	Comprehensive training: 4 h of theoretical instruction, 4 h of pig‐eye wet lab, 4 h of physician observation, 20 preparations, 100 supervised patient injections, and final competency assessment. Continuous auditing; physicians retained overall patient responsibility	Survey; high satisfaction, reduced waiting time valued
Varma et al. (2013)	A total of 20 postoperative complications were observed among 1400 injections (14.3 per 1000), with no cases of endophthalmitis	Implemented via a structured model including approval, training, assessment, and supervised to independent practice. Consultants retained clinical responsibility; nurses held procedural responsibility with prompt access to medical support	Structured training: didactic courses, practical exercises (including wet lab), 25 successful injections required for certification. Stepwise supervision by consultant ophthalmologist with ongoing auditing and supervision	Survey and pain score; high satisfaction reported

Abbreviation: NR, not reported.

### 3.1 Safety

Reported safety outcomes varied slightly between countries and study designs, but all remained within established safety ranges for intravitreal injection procedures. Endophthalmitis occurred rarely across all studies (0%–0.07%), corresponding to approximately 25 cases among more than 67 000 injections (Ahmed et al., 2022; Bolme et al., 2020; DaCosta et al., 2014; Hasler et al., 2015; Samalia et al., 2016; Simcock et al., 2014; Teo et al., 2020). In United Kingdom studies, reported rates ranged between 0.015% and 0.04%. No study reported a higher occurrence when injections were administered by nurses compared with physicians (Ahmed et al., 2022; Bolme et al., 2020; Hasler et al., 2015; Simcock et al., 2014).

Chakouri et al. (2017) reported no adverse events following the introduction of nurse‐administered injections in Denmark (Chakouri et al., 2017). In the Norwegian randomized controlled trial by Bolme et al. (2020), no sight‐threatening adverse events were reported (Bolme et al., 2020).

Minor, transient events were infrequent and within expected ranges for intravitreal injection procedures. Subconjunctival haemorrhage was reported in 0.3%–4.0% of nurse‐administered injections and transient increases in intraocular pressure occurred in <1% of cases (Ahmed et al., 2022; Michelotti et al., 2014; Samalia et al., 2016; Teo et al., 2020; Varma et al., 2013). Corneal abrasions were rare (<0.2%) and no study reported vision‐threatening consequences (Ahmed et al., 2022; Michelotti et al., 2014; Teo et al., 2020). Across all implementation contexts, complication rates were reported within acceptable limits for intravitreal therapy (Ahmed et al., 2022; Bolme et al., 2020; DaCosta et al., 2014; Hasler et al., 2015; Simcock et al., 2014).

### 3.2 Implementation

Implementation of nurse‐administered intravitreal injection services was reported across multiple countries, typically initiated to meet increasing demand for anti‐VEGF treatment within ophthalmology services (Bolme et al., 2020; DaCosta et al., 2014; Hasan et al., 2020; Michelotti et al., 2014; Varma et al., 2013). In the United Kingdom, DaCosta et al. (2014) reported that the initiative began as a pilot project requiring approval from the hospital's legal department and ongoing audits under consultant supervision. Governance approval and clear delineation of responsibilities were emphasized as prerequisites before nurses could practice independently (DaCosta et al., 2014). Gallagher (2017) reported implementation aligned with national and local guidelines, supported by master's‐level training and continuous audit (Gallagher, 2017). At the West of England Eye Unit (Royal Devon and Exeter NHS Foundation Trust), Simcock et al. (2014) described a phased implementation under oversight from the Clinical Governance Committee to ensure risk management and audit reporting (Simcock et al., 2014). Michelotti et al. (2014) and Hasan et al. (2020) both documented rapid expansion of nurse injector roles once institutional approval and clear protocols were established; the latter reported that use of the Precivia® assist device standardized patient positioning and workflow (Hasan et al., 2020; Michelotti et al., 2014).

In Denmark, Chakouri et al. (2017) described the interdisciplinary implementation involving collaboration between clinical management, nursing management and physicians to develop a structured training programme (Chakouri et al., 2017). Hasler et al. (2015) introduced a combined model in which both resident ophthalmologists and trained nurses performed injections under standardized protocols in the operating theatre (Hasler et al., 2015). In Norway, implementation was conducted through structured projects. Bolme et al. (2020) reported task shifting as part of a randomized controlled trial integrated in daily practice, with a physician always present. Subsequent publications (Bolme et al., 2021, 2023) described the development of a 10‐day training and certification programme, the appointment of a dedicated medical lead, and annual re‐certification. Nurses highlighted the importance of managerial support and clear role definition as factors that supported implementation (Bolme et al., 2020, 2021, 2023). In New Zealand, Samalia et al. (2016) reported that the nurse injector scheme was implemented in parallel with physician‐led clinics to maintain patient safety during the initial phase (Samalia et al., 2016). Senior ophthalmologists retained clinical responsibility while nurses progressively assumed up to 84% of injections after two years (Samalia et al., 2016). In Singapore, Teo et al. (2020) described implementation at a national tertiary centre involving university‐trained nurses working closely with management and senior ophthalmologists (Teo et al., 2020). Organizational adjustments such as dedicated treatment rooms, parallel physician availability and comprehensive patient education were reported as part of the implementation process (Teo et al., 2020). Across all settings, implementation was consistently reported to involve formal approval processes, managerial support, appointment of a responsible ophthalmologist and use of audit mechanisms (Bolme et al., 2020; Bolme et al., 2021; DaCosta et al., 2014; Gallagher, 2017; Hasan et al., 2020; Michelotti et al., 2014; Samalia et al., 2016; Simcock et al., 2014; Teo et al., 2020).

### 3.3 Training and supervision

Training and supervision were formally structured and competence‐based across all studies establishing nurse‐led intravitreal injection services. Programmes typically combined theoretical instruction, wet lab training on porcine or model eyes, direct observation of physician‐led procedures and a defined number of supervised injections before certification for independent practice (Ahmed et al., 2022; Bolme et al., 2020; Chakouri et al., 2017; DaCosta et al., 2014; Gallagher, 2017; Hasan et al., 2020; Hasler et al., 2015; Michelotti et al., 2014; Samalia et al., 2016; Simcock et al., 2014; Teo et al., 2020; Varma et al., 2013). In the United Kingdom, DaCosta et al. (2014) described a structured, multi‐step programme comprising a one‐day course (theory and wet lab training on porcine eyes), observation of ≥20 procedures, and at least 100 supervised injections before certification (DaCosta et al., 2014). Continuous professional development and access to a supervising consultant were required. Michelotti et al. (2014) implemented an intensive programme in which nurses observed more than 1000 procedures, performed the first 200 under direct supervision and completed a final competency assessment prior to independent practice (Michelotti et al., 2014). Gallagher (2017) reported that master's‐trained nurses completed 50 observed and 50 supervised procedures and participated in annual audits (Gallagher, 2017). Hasan et al. (2020) described a 12‐week training programme with theoretical and practical components under continuous supervision; injections were performed using the Precivia® device under physician oversight (Hasan et al., 2020). Simcock et al. (2014) described one‐to‐one training with a vitreoretinal surgeon, requiring at least 20 supervised procedures before independent practice (Simcock et al., 2014). In Denmark, Hasler et al. (2015) reported a stepwise approach including model training and 8–10 supervised injections (Hasler et al., 2015). Chakouri et al. (2017) described a structured programme consisting of a six‐hour theoretical module, practical training on model eyes and four weeks of clinical supervision before certification (Chakouri et al., 2017). In Norway, implementation was carried out as a structured project. Bolme et al. (2020) reported the task shifting as part of a randomized controlled trial, in which nurses were certified for independent practice following evaluation by an external retinal surgeon, with ophthalmologist support available at all times (Bolme et al., 2020). The structured training programme lasted 10 days and included workshops, wet lab exercises, and observation (Bolme et al., 2021). The nurses also introduced annual re‐certification on their own initiative to ensure quality (Bolme et al., 2021), although economic evaluations were based on biennial re‐certification (Bolme et al., 2023). In New Zealand, nurses completed a standardized training program with ≥50 observed or supervised injections before independent practice (Samalia et al., 2016). In Singapore, the training comprised four hours of theory, four hours of wet lab training on porcine eyes, four hours of observation, 20 preparatory exercises and 100 supervised patient injections followed by a final competency assessment (Teo et al., 2020).

### 3.4 Patient‐reported experience

Across all studies, patients consistently reported high satisfaction and acceptance of nurse‐led treatment, often comparable to physician‐administered services. In the United Kingdom, DaCosta et al. (2014) found that patients were satisfied with the safety and professionalism of nurse‐administered injections, although a minority declined the treatment in the pilot phase, citing preference for a doctor or anxiety related to lack of nurse experience (DaCosta et al., 2014). Michelotti et al. (2014) and Gallagher (2017) reported very high satisfaction, primarily due to shorter waiting times, improved accessibility and empathetic communication. In Gallagher (2017), 100% of patients were satisfied; 81% were indifferent to the profession of the injector, while 15% preferred a nurse (Gallagher, 2017; Michelotti et al., 2014). However, a minority explicitly stated a preference for a doctor‐administered injection, citing concerns regarding nurse experience and perceived ability to manage acute complications (Gallagher, 2017). Hasan et al. (2017, 2020) found that patients experienced lower anxiety and high levels of trust in nurses, with consistently high satisfaction across more than 30 000 injections (Hasan et al., 2017; Hasan et al., 2020). Varma et al. (2013) reported that patients reported comfort levels comparable to physician‐administered treatment and expressed high satisfaction with nurse‐delivered injections. No patients refused nurse treatment, and continuity of care was highlighted as a positive aspect (Varma et al., 2013). In Denmark, Chakouri et al. (2017) reported no patient concerns or refusals; patients expressed trust in nurses' competence and appreciated improved access to treatment (Chakouri et al., 2017). In New Zealand, Samalia et al. (2016) reported high patient acceptance and feelings of reassurance, attributed to clear communication and standardized procedures (Samalia et al., 2016). In Singapore, Teo et al. (2020) reported high satisfaction, with patients highlighting reduced waiting time and efficient patient flow as advantages (Teo et al., 2020).

Despite limited qualitative data, all studies consistently reported high patient satisfaction and no negative experiences or refusals of nurse‐administered intravitreal injections.

## 4 DISCUSSION

This systematic review synthesized evidence from 16 studies comprising 100 150 intravitreal injections delivered by specially trained ophthalmic nurses (Ahmed et al., 2022; Bolme et al., 2020; Bolme et al., 2021; Bolme et al., 2023; DaCosta et al., 2014; Gallagher, 2017; Hasan et al., 2017; Hasan et al., 2020; Hasler et al., 2015; Michelotti et al., 2014; Samalia et al., 2016; Simcock et al., 2014; Teo et al., 2020; Varma et al., 2013). Across study designs and settings, nurse‐administered injections were consistently found to be as safe and effective as those delivered by physicians. Moreover, these services substantially increased treatment capacity while maintaining safety (Ahmed et al., 2022; Bolme et al., 2020; DaCosta et al., 2014; Hasan et al., 2020; Michelotti et al., 2014; Simcock et al., 2014; Teo et al., 2020). Rates of serious complications, including endophthalmitis, were very low (~0.04%), patient satisfaction was high, and structured training programmes were commonly described (DaCosta et al., 2014; Simcock et al., 2014; Teo et al., 2020; Varma et al., 2013). Several studies reported significant gains in capacity when nurses were assigned the majority of injections ranging from 25% to 85% of total injections after full implementation (Ahmed et al., 2022; Bolme et al., 2020; DaCosta et al., 2014; Hasan et al., 2020; Michelotti et al., 2014; Samalia et al., 2016; Simcock et al., 2014; Teo et al., 2020).

The incidence of endophthalmitis across the included studies was approximately 0.04%, fully comparable to rates reported in large physician‐led cohorts (0.02%–0.05%) (Hasler et al., 2015; Simcock et al., 2014). Importantly, no study suggested an increased risk of complications when injections were performed by non‐physicians. These findings confirm and extend the results of the earlier systematic review by Rasul et al. (2016), which concluded that non‐physician‐delivered intravitreal injections are feasible and safe (Rasul et al., 2016). Our inclusion of newer data from Nordic and Asian contexts strengthens the external validity of these conclusions (Bolme et al., 2020; Bolme et al., 2021; Bolme et al., 2023; Teo et al., 2020). The inclusion of more recent evidence from Norway (Bolme et al., 2020; Bolme et al., 2021; Bolme et al., 2023), Singapore (Teo et al., 2020) and New Zealand (Samalia et al., 2016) further supports this conclusion. Low complication rates likely reflect rigorous aseptic technique, competency‐based training and clear governance frameworks that ensure procedural consistency and oversight. Other serious complications such as retinal detachment, lens injury and vitreous haemorrhage were rarely or never reported (DaCosta et al., 2014; Hasler et al., 2015; Samalia et al., 2016; Simcock et al., 2014).

The study by Bolme et al. (2020), a randomized single‐masked non‐inferiority trial (RCT), concluded that nurse‐administered intravitreal injections were non‐inferior to physician‐administered injections regarding the maintenance of best‐corrected visual acuity (BCVA) after one year (Bolme et al., 2020). In the RCT, which included a total of 2077 injections during the study period, three ocular adverse events were recorded, and no sight‐threatening complications such as lens damage or retinal detachment were observed. However, it is crucial to note the study's limitations regarding the assessment of rare but severe complications, such as endophthalmitis. The incidence of severe complications in the literature is typically low, often in the range of 1–10 per 10 000 injections (corresponding to 0.01% to 0.1%) (Hasler et al., 2015; Teo et al., 2020). Since the RCT only encompassed approximately 2000 injections in total, the study's sample size is insufficient to statistically detect or refute differences in such rare complications between the nurse and physician groups. Therefore, the absence of a difference in complication rates in this trial is expected and should not be interpreted as definitive evidence of equivalent safety, but rather as an indication that larger observational cohorts are required to assess rare events. In the RCT, the incidence of endophthalmitis was 0.05% per injection. This finding is consistent with rates reported in contemporary large‐scale studies of Intravitreal anti‐VEGF therapy, where the incidence typically ranges between 0.02% and 0.08% per injection (Bolme et al., 2020; Hasler et al., 2015).

Minor adverse events, such as subconjunctival haemorrhage or transient intraocular pressure (IOP), occurred at rates of 0.1%–6%, consistent with those seen in physician‐led services (Ahmed et al., 2022; DaCosta et al., 2014; Samalia et al., 2016; Simcock et al., 2014). None of these events required additional intervention beyond standard management. However, the available evidence provides limited insight into the safety of nurse‐administered injections in more complex patient populations. One study explicitly excluded patients with complicating factors such as nystagmus, agitation, severe systemic comorbidities or reduced cooperation, and few provided data on how such cases were managed in practice (Hasler et al., 2015). As a result, the current evidence base primarily reflects low‐risk patients in controlled clinical settings. Future studies should therefore investigate the safety and feasibility of nurse‐administered intravitreal injections in higher‐complexity populations to determine the generalizability of current findings beyond standard candidates for anti‐VEGF therapy.

Overall, findings from eight cohorts and one randomized controlled trial confirm that intravitreal injections performed by competency‐assessed nurses are a safe and effective clinical practice, provided that standardized training, aseptic protocols and continuous audit are maintained.

Implementation of nurse‐led intravitreal injection services consistently demonstrated high feasibility and sustainability across healthcare systems (Bolme et al., 2020; DaCosta et al., 2014; Michelotti et al., 2014; Samalia et al., 2016; Teo et al., 2020). Most programmes were embedded within existing ophthalmology departments, where nurses gradually assumed responsibility for the majority of anti‐VEGF injections once fully trained and certified (Ahmed et al., 2022; DaCosta et al., 2014; Samalia et al., 2016; Simcock et al., 2014). In the United Kingdom, nurses performed over 80% of all injections once services were established, while in Denmark, nurses and physicians‐in‐training together delivered more than 38 000 injections during a five‐year period (Ahmed et al., 2022; Bolme et al., 2020; Hasler et al., 2015; Michelotti et al., 2014; Simcock et al., 2014). Across settings, implementation resulted in shorter waiting times, reduced physician workload and lower per procedure costs (Ahmed et al., 2022; Bolme et al., 2020; DaCosta et al., 2014; Simcock et al., 2014; Teo et al., 2020). In Singapore, nurse‐led injection delivery reduced average waiting times from approximately 35 minutes to four minutes, while ophthalmologists' time was freed for surgery and complex diagnostics (Teo et al., 2020). Similarly, in Norway, a health‐economic analysis demonstrated a cost saving of €5.5 per injection, corresponding to an annual hospital saving of €43 000–49 000 (Bolme et al., 2023). These outcomes align with WHO's task‐shifting principles, demonstrating how advanced nursing roles can alleviate workforce shortages without compromising quality or safety (Ahmed et al., 2022; Bolme et al., 2020; Hasler et al., 2015; Michelotti et al., 2014; World Health Organization et al., 2008). Although the reported cost saving of €5.5 per injection may appear modest, it should be interpreted in the context of high‐volume treatment delivery, resulting in substantial annual savings at the institutional level. Moreover, the primary economic benefit of nurse‐led injection services lies not in direct cost reduction per procedure, but in improved workforce flexibility, increased clinical capacity and enhanced access to timely treatment (Bolme et al., 2023). Taken together, these findings suggest that nurse‐led injection services not only maintain safety but also enhance clinical efficiency, patient flow and long‐term sustainability. The success of implementation relied on strong institutional support, clear governance structures and ongoing audit systems that ensured accountability and continuous quality improvement.

Across diverse settings, training models used a structured, competency‐based approach, though the content and duration varied by country. In the United Kingdom, training programmes typically required between 25 and 200 supervised injections before nurses were permitted to practice independently (DaCosta et al., 2014; Michelotti et al., 2014; Varma et al., 2013). In Singapore, Teo et al. (2020) described a structured curriculum including classroom teaching, wet lab training, observation of 20 physician‐administered injections, followed by 100 supervised procedures (Teo et al., 2020). In New Zealand, Samalia et al. (2016) required approximately 50 supervised injections, whereas in Norway, Bolme et al. (2020) adopted a formal stepwise training framework as part of a randomized controlled trial and subsequent qualitative evaluation (Bolme et al., 2020; Bolme et al., 2021; Bolme et al., 2023; Samalia et al., 2016). Despite these differences, all programmes followed the same principle: structured, competency‐based training with close supervision and formal assessment prior to independent practice. These findings indicate that competence assurance, rather than professional title, is the critical determinant of procedural safety. Variability in training duration and structure likely reflects differences in governance frameworks, local regulations and pre‐existing ophthalmic expertise among nurses. Collectively, the evidence supports that rigorous, standardized education and supervision are essential components of safe task shifting in ophthalmic care.

It is important to acknowledge that while these training frameworks consistently support safe clinical performance, there is a notable academic gap in the published literature regarding the scientific validity and pedagogical effectiveness of the training modalities themselves (Bolme et al., 2020). Success is typically evaluated based on low complication rates and demonstrated non‐inferiority, yet no studies have formally randomized or compared different educational interventions such as duration, content or instructional format to determine the most effective model for achieving and maintaining procedural competence (Bolme et al., 2020).

Nurses in a Norwegian study described experiencing moments of uncertainty, even though they generally felt competent (Bolme et al., 2021). This uncertainty primarily arose from the increased level of clinical responsibility, including the fear of making mistakes, as well as difficulty assessing contraindications such as blepharitis, which frequently required medical consultation (Bolme et al., 2021). Some nurses also reported feeling discouraged when patients asked complex diagnostic or prognostic questions beyond their professional scope, describing this as “taking away some of the pride” they otherwise experienced in running the clinic (Bolme et al., 2021). This uncertainty reflects a broader unresolved debate within the field: should nurses be limited to administering the injection itself, or should their role also include diagnosis, therapeutic decision‐making and modification of treatment regimens? In most of the included studies, nurses were responsible solely for the administration of injections, while all clinical decision‐making remained under the authority of an ophthalmologist. The need for sustained competence over time is further emphasized by nurses who explicitly requested “continuous learning” and “regular assessment of injection technique” to prevent the task from “quickly insufficient to ensure long‐term competence and must be reinforced through structured governance and ongoing re‐training a re‐certification” (Bolme et al., 2021).

Studies from the United Kingdom and Singapore reported high levels of patient satisfaction and confidence in nurse‐led intravitreal injection services, in some cases exceeding those reported for physician‐administered procedures. In an evaluation from Swindon, United Kingdom, 76.9% of patients strongly agreed that the nurse was more empathetic and attentive to their needs compared with prior physician‐led experiences (Hasan et al., 2017; Hasan et al., 2020) Patients also reported feeling safe when injections were administered by nurses. In Singapore, nurse‐delivered treatment scored higher than physician‐delivered injections in five out of seven satisfaction domains, including overall satisfaction, interpersonal conduct and perceived competence (Teo et al., 2020) Across studies, patient satisfaction was consistently high; however, these findings should be interpreted with methodological caution, as the underlying evaluations were almost exclusively based on non‐validated Likert scale questionnaires without blinding or independent data collection, introducing a risk of response and social desirability bias. In Gallagher (2017), 81% of patients were indifferent to whether the injection was delivered by a nurse or a doctor, while 15% preferred a nurse and only 4% preferred a doctor (Gallagher, 2017). The minority who favoured a doctor cited concerns about the nurse's clinical experience and perceived ability to manage acute complications (Gallagher, 2017). This suggests that acceptance of nurse‐led intravitreal injection services depends not only on demonstrated clinical safety but also on patients' perception of professional authority and ability to respond to complications, particularly in early implementation phases.

A randomized study by Mohamed et al. (2018) used a modified version of the validated patient questionnaire to compare patient experiences with intravitreal injections administered by a specially trained nurse versus a senior physician. The study found no significant differences in patient satisfaction or pain perception between the groups, supporting the acceptability and perceived safety of nurse‐led care (Mohamed et al., 2018). Future research should therefore employ validated patient‐reported outcome measures and explore deeper constructs such as trust, perceived safety and expectations regarding professional responsibility, rather than relying solely on overall satisfaction metrics.

Limitations of this systematic review should be considered when interpreting its findings. Although the review is strengthened by a systematic search strategy and a structured, domain‐based synthesis, its conclusions are constrained by the limitations of the available evidence. Only a modest number of studies met the inclusion criteria, and substantial heterogeneity in study design, methodology and outcome reporting prevented formal meta‐analysis. Moreover, most studies were observational service evaluations with retrospective or non‐comparative designs, which limits the certainty and generalizability of the findings. Furthermore, most studies did not directly compare nurse‐ and physician‐administered injections, limiting conclusions about relative safety. These limitations primarily reflect the early‐stage maturity of the research field rather than weaknesses in the review process itself.

## 5 CONCLUSION

This systematic review demonstrates that nurse‐led intravitreal injection services, when supported by structured training and governance, can be delivered safely and efficiently. The model offers a scalable strategy to address the growing demand for anti‐VEGF therapy and may alleviate pressure on overstretched medical retina services. However, the current evidence base remains limited. Most available studies are single‐centre, observational and inadequately powered to detect rare adverse events or long‐term outcomes. High‐quality, multicentre randomized controlled trials are therefore urgently needed, ideally combined with health‐economic analyses and validated patient‐reported outcome measures. Future research should also compare alternative workforce models including nurses, optometrists and orthoptists and evaluate different training and certification frameworks to inform internationally applicable standards.

## FUNDING INFORMATION

This study is funded by a grant (VEL75910) from VELUX FONDEN.

## Supporting information


**File S1.** Search strategy. PRISMA 2020 flow diagram for new systematic reviews which included searches of databases and registers only.
