# Supplementary material for: Non‐physician delivered intravitreal injection service: A systematic review of safety, implementation, training and patient experience
Source: Acta Ophthalmol. 2026 Feb 11;104(6):e615–29. doi: 10.1111/aos.70104 (PMC13460359; doi:10.1111/aos.70104)
Supplement: Supplementary file 1 [file AOS-104-e615-s001.docx]

**Supplementary File 1. Search Strategy**

**Title of the systematic review**
Task-shifting of intravitreal injections from physicians to non-physician healthcare professionals: a systematic review

**Date of search:**
14 September 2025

**Databases searched:**
PubMed, Embase, Cochrane Central, BIOSIS Previews, Current Contents Connect, Data Citation Index, Derwent Innovations Index, KCI–Korean Journal Database, Preprint Citation Index, ProQuest™ Dissertations & Theses Citation Index, SciELO Citation Index, Web of Science Core Collection, and CINAHL.

**Search conducted by:**
Yousif Subhi (Y.S.), trained researcher

**Documentation of the literature search across databases:**

**PubMed:**

**Cochrane Central:**

```
Search Name:
Date Run:	15/09/2025 00:33:10
Comment:

ID	Search	Hits
#1	nurse*	42666
#2	orthoptist*	75
#3	optometrist*	425
#4	non-physician*	237
#5	intravitreal*	5032
#6	intra-vitreal*	48
#7	#1 OR #2 OR #3 OR #4	43237
#8	#5 OR #6	5038
#9	#7 AND #8	32
```

**EMBASE:**

```
Embase <1974 to 2025 September 11>

1	nurse*.mp. [mp=title, abstract, heading word, drug trade name, original title, device manufacturer, drug manufacturer, device trade name, keyword heading word, floating subheading word, candidate term word]	545174
2	nurse/	173985
3	orthoptist*.mp. [mp=title, abstract, heading word, drug trade name, original title, device manufacturer, drug manufacturer, device trade name, keyword heading word, floating subheading word, candidate term word]	815
4	orthoptist/	282
5	optometrist*.mp. [mp=title, abstract, heading word, drug trade name, original title, device manufacturer, drug manufacturer, device trade name, keyword heading word, floating subheading word, candidate term word]	5415
6	optometrist/	2027
7	non-physician*.mp. [mp=title, abstract, heading word, drug trade name, original title, device manufacturer, drug manufacturer, device trade name, keyword heading word, floating subheading word, candidate term word]	3176
8	intravitreal*.mp. [mp=title, abstract, heading word, drug trade name, original title, device manufacturer, drug manufacturer, device trade name, keyword heading word, floating subheading word, candidate term word]	47964
9	intra-vitreal*.mp. [mp=title, abstract, heading word, drug trade name, original title, device manufacturer, drug manufacturer, device trade name, keyword heading word, floating subheading word, candidate term word]	370
10	intravitreal drug administration/	9688
11	1 or 2 or 3 or 4 or 5 or 6 or 7	553375
12	8 or 9 or 10	48087
13	11 and 12	164
```

**Web of Science Core Collection, Biological Abstracts, BIOSIS Citation Index, BIOSIS Previews, Current Contents Connect, Data Citation Index, Derwent Innovations Index, Grants Index, KCI-Korean Journal Database, Policy Citation Index, ProQuest ™ Dissertations & Theses Citation Index, SciELO Citation Index:**

**CINAHL:**
